# Programmed Deviations of Ribosomes From Standard Decoding in Archaea

**DOI:** 10.3389/fmicb.2021.688061

**Published:** 2021-06-04

**Authors:** Federica De Lise, Andrea Strazzulli, Roberta Iacono, Nicola Curci, Mauro Di Fenza, Luisa Maurelli, Marco Moracci, Beatrice Cobucci-Ponzano

**Affiliations:** ^1^Institute of Biosciences and BioResources – National Research Council of Italy, Naples, Italy; ^2^Department of Biology, University of Naples Federico II, Complesso Universitario di Monte S. Angelo, Naples, Italy; ^3^Task Force on Microbiome Studies, University of Naples Federico II, Naples, Italy

**Keywords:** alpha-fucosidase, recoding, frameshifting, pyrrolysine, selenocysteine, archaea

## Abstract

Genetic code decoding, initially considered to be universal and immutable, is now known to be flexible. In fact, in specific genes, ribosomes deviate from the standard translational rules in a programmed way, a phenomenon globally termed recoding. Translational recoding, which has been found in all domains of life, includes a group of events occurring during gene translation, namely stop codon readthrough, programmed ± 1 frameshifting, and ribosome bypassing. These events regulate protein expression at translational level and their mechanisms are well known and characterized in viruses, bacteria and eukaryotes. In this review we summarize the current state-of-the-art of recoding in the third domain of life. In Archaea, it was demonstrated and extensively studied that translational recoding regulates the decoding of the 21st and the 22nd amino acids selenocysteine and pyrrolysine, respectively, and only one case of programmed –1 frameshifting has been reported so far in *Saccharolobus solfataricus* P2. However, further putative events of translational recoding have been hypothesized in other archaeal species, but not extensively studied and confirmed yet. Although this phenomenon could have some implication for the physiology and adaptation of life in extreme environments, this field is still underexplored and genes whose expression could be regulated by recoding are still poorly characterized. The study of these recoding episodes in Archaea is urgently needed.

## Introduction

Translation, in its basic mechanism, is universally conserved and is performed by one of the most complex and sophisticated cell machineries, the ribosomes, in which the majority of protein components are highly conserved in all of the domains of life. However, both the genetic code and its decoding are neither universal nor immutable due to the complex nature of translation. The genetic code is not quite universal; in fact, it is well established that the meaning of some codons in certain organelles and organisms has been reassigned (codon reassignment) for all the mRNAs belonging to that organelle or organism. Unlike codon reassignment, in non-canonical translation mechanisms the alteration of the translation rules does not occur for the whole organism but is limited only to specific genes and often correlated to particular physiological conditions that regulate their translation. The discovery of these gene expression regulatory mechanisms has completely changed our view of the disrupted genes that are often found during genome sequencing. In fact, sequenced genomes often reveal interrupted coding sequences and they are generally considered sequencing errors or pseudogenes. It is now well known that the majority of these interrupted genes are functional and encode proteins whose expression is regulated. Non-canonical translation mechanisms have been identified in all steps of the translation: initiation, elongation and termination. Well known strategies related to the initiation of translation are internal ribosome entry, leaky scanning, non-AUG initiation, ribosome shunting and reinitiation. These strategies are used extensively by viruses, presumably providing alternative ways to express different proteins from a single mRNA, facilitating the access to overlapping ORFs and overcoming the structural differences present in viral transcripts in comparison with cellular mRNAs. Furthermore, it has been shown that cancer cells exploit these alternative modes of translation initiation for their survival and proliferation under stressful conditions (for comprehensive reviews see Firth and Brierley, 2012; Pooggin and Ryabova, 2018; Sriram et al., 2018; Yang and Wang, 2019; Cao and Slavoff, 2020).

Programmed deviations from the standard translational rules occuring during translational elongation or termination steps are termed *recoding* (Gesteland and Atkins, 1996; Firth and Brierley, 2012) and, often in competition with standard decoding, have crucial roles in the regulation of gene expression (Baranov et al., 2002). These universal mechanisms are +*1* or *–1 programmed frameshifting* (PRF) and *ribosome hopping*, which occur during the elongation step, and *stop codon readthrough*/*redefinition* occurring during the termination step (Farabaugh, 1996; Gesteland and Atkins, 1996; Baranov et al., 2002; Namy et al., 2004; Ling et al., 2015; Atkins et al., 2016; Rodnina et al., 2020).

**FIGURE 1:**
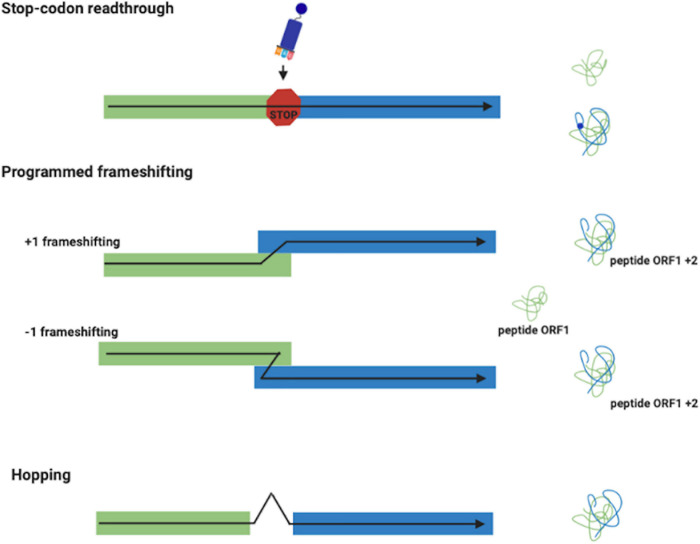
Recoding events. Stop-codon readthrough: a different meaning is assigned to a stop codon with the insertion of the unusual amino acids selenocysteine and pyrrolysine. Frameshifting (+1 and –1): produces two polypeptides from different reading frames of the same mRNA. Ribosome Hopping: synthesizes one protein from two open discontinuous reading frames.

In *stop codon readthrough* (Figure 1), the termination codon is decoded by a tRNA rather than a release factor, allowing ribosomes to synthesize an extended polypeptide. In specific genes, this tRNA carries the unusual amino acids selenocysteine (Hatfield and Gladyshev, 2002) or pyrrolysine (Namy et al., 2004), and specific stimulatory elements downstream to the stop codon regulate this process (Bertram et al., 2001). In PRF (Figure 1), ribosomes are induced to switch, upward or backward for +1 and –1 PRF, respectively, to an alternative, overlapping reading frame at a specific shift site (Farabaugh, 1996; Atkins et al., 2016). This is a regulated process and its frequency depends by genes and on the presence of stimulatory signals in the mRNA. PRF has been detected in organisms from all three domains of life, but it is very common in viruses (Baranov et al., 2006; Firth and Brierley, 2012), in which several recoding events have been described and characterized. R*ibosome hopping* (Figure 1) is a rarer recoding event in which the ribosome stops in a precise site of the mRNA and re-starts translation downstream bypassing few nucleotides. This mechanism has been discovered and studied in detail in the gene 60 of bacteriophage T4 (Herr et al., 2004). Ribosomal bypass occurs at *hop* elements where the ribosome block at the “take-off codon,” immediately upstream of a stop codon followed by a hairpin, determining the dissociation of the peptidyl-tRNA which re-associates at the “landing triplet,” 50 nt downstream, where the translation resumes. More recently, several bypassing elements (*byps*) have been reported in *Magnusiomyces capitatus* mitochondria, suggesting that hopping is more frequent than previously thought (Lang et al., 2014; Nosek et al., 2015). An updated list of genes regulated by recoding can be found in the Recode2 database (^1^ Bekaert et al., 2010).

In recent years, bioinformatic analyses of sequenced genomes available in databases have allowed the identification of numerous interrupted genes that could be potential candidates for genes whose expression is regulated by recoding (van Passel et al., 2007; Cobucci-Ponzano et al., 2010; Sharma et al., 2011). However, to date, most of these have been identified serendipitously. A huge boost to the study of non-canonical translation mechanisms came from the development of ribosome profiling, or ribo-seq, a technique that provides genome-wide information on protein synthesis (GWIPS) *in vivo* (Ingolia et al., 2009). Ribosome profiling is based on the deep sequencing of ribosome-protected mRNA fragments and the high resolution of this technique allows the determination of ribosome density along individual cellular mRNA molecules. The real power of ribosome profiling is in its ability to obtain position-specific information regarding ribosome locations on mRNAs, allowing the identification of unpredictable non-canonical translation events. Since its invention, the ribosome profiling technique has been applied in a range of studies in both prokaryotic and eukaryotic organisms, but only one analysis is reported in Archaea (Michel and Baranov, 2013; Brar and Weissman, 2015; Gelsinger et al., 2020).

In Archaea, non-canonical translation events have been demonstrated only during the elongation and the termination steps. In particular, termination codon readthrough events regulating the incorporation of the amino acids selenocysteine and pyrrolysine (Nicholas et al., 2018; Rother and Quitzke, 2018), and –1 PRF allowing the expression of a functional α-L-fucosidase (Cobucci-Ponzano et al., 2003a,b, 2005a, 2005b, 2006, 2012). More recently, –1 PRF was also reported in siphoviruses tailed virus 1 (HVTV-1) and three viruses (HCTV-1,2 and 5) that infect halophilic archaea (Pietila et al., 2013; Sencilo et al., 2013). Increasing evidence suggests that the flexibility of genetic code decoding is a trait selected during evolution to benefit microorganisms under certain physiological conditions, increasing their fitness (Ling et al., 2015). This could be particularly relevant for Archaea, often inhabiting extreme environments in which changes in nutrients, pH, temperatures, etc. are rather common and occur rapidly and reversibly, and may expose microbes to the necessity to modify reversibly gene expression through quick mechanisms (Iacono et al., 2020; Onofri et al., 2020; Strazzulli et al., 2020). Here, we summarize the current state of the art on the studies on the mechanisms of translational recoding found in Archaea, often living in extreme conditions, to provide an update of this interesting and relatively unknown mechanism of regulation of gene expression in the third domain of life.

## Stop Codon Readthrough

In stop codon readthrough it is important to distinguish between two different mechanisms: ‘reassignment’ and ‘recoding’ (Atkins and Baranov, 2010). In codon reassignment, occurring for example in certain mitochondria (Barrell et al., 1979; Osawa et al., 1992), the meaning of particular codons is always reassigned. That codon has only the new meaning and this redefinition is context-independent. These reassignments mainly involve UAG or UGA codons encoding an amino acid instead of a termination signal. Instead, in context-dependent codon redefinition, such event only applies to particular stop codons. Stop codon readthrough is dynamic, with the new definition competing with the standard one, so only a part of the product reflects the new meaning. When it occurs, this redefinition mechanism is a recoding event (Figure 1) in which UAG or UGA specify for the amino acids selenocysteine and pyrrolysine, respectively.

### The 21st Amino Acid: Selenocysteine

The twenty-first amino acid selenocysteine (Sec) contains selenium, an essential micronutrient for many organisms, and is translationally incorporated into proteins in Bacteria, Eukarya and Archaea (for a comprehensive review see Ambrogelly et al., 2007). Sec does not have a fully dedicated codon, but it is inserted in response to the UGA stop codons that are recoded in the presence of specific regulation signals *in cis*. When translating ribosomes encounter an UGA stop codon in the presence of regulative signals, they are loaded with a specific Sec-tRNA, promoting the insertion of a Sec residue in this location. In fact, in response to those signals, a Sec-specific elongation factor (SelB) replaces the standard EF-Tu uniquely for the translation of Sec UGA codons and recruits the specific Sec-tRNA (see below for the description of the mechanism of insertion). In bacteria, bSelB is homologous in the N-terminal part to the standard elongation factor ET-Tu, while it has a C-terminal extension responsible for binding to SECIS elements. In contrast to that, the C-terminal extension of the archaeal aSelB is shorter and unrelated to that of bacteria and these structural features are conserved in the eukaryotic homolog eSelB (Kromayer et al., 1996; Fagegaltier et al., 2000; Tujebajeva et al., 2000; Yoshizawa et al., 2005).

This structural difference is most likely the cause of the lack of binding of aSelB to a cognate SECIS element *in vitro* (Rother et al., 2000).

The presence of Sec as selenium carrier in natural proteins, called selenoproteins, was first demonstrated in clostridial glycine reductase (Cone et al., 1976). Sec was then found in enzymes maintaining cell redox balance defending the cell against reactive oxygen species. In humans, the selenoproteome comprises 25 members, whose biological functions have been implicated in diverse human diseases ranging from cardiovascular and endocrine disorders to abnormalities in immune responses and cancer (Bellinger et al., 2009).

Selenoproteins are often enzymes with oxidoreductase function in which Sec is the catalytic redox active site. Homologs proteins in which Sec is replaced with cysteine (Cys) exist for the great majority of selenoproteins, although they perform the same reaction less efficiently (Fomenko and Gladyshev, 2012). It is generally accepted that Sec is used in place of Cys due to its higher reactivity, which leads to improved catalytic efficiency, although the exchangeability of Sec and Cys is debated (Gromer et al., 2003; Castellano, 2009; Hondal and Ruggles, 2011; Hondal et al., 2013). The fact that the Sec-containing proteins are more active if compared to the Cys-containing versions was elegantly demonstrated by inactivating the Sec-specific elongation factor SelB in *M. maripaludis* JJ and observing that this led to overexpression of Cys-containing versions of selenoproteins (Rother et al., 2003). Selenoproteins are not present in all organisms but their distribution is scattered among all the three domains of life in which, however, they perform different functions (Mariotti et al., 2015). In Bacteria, selenoproteins are involved in redox homeostasis, electron transport/energy metabolism, compound detoxification, and oxidative protein folding. In contrast, in Archaea they are involved in methanogenesis, with the only exception of selenophosphate synthetase (SPS), involved in Sec biosynthesis (Stock and Rother, 2009; Rother and Krzycki, 2010). In Eukarya, selenoproteins are mainly involved in redox regulation, antioxidant defense, protein repair, and oxidative protein folding, with very few examples involved in compound detoxification, electron transport, and energy metabolism (Labunskyy et al., 2014). However, Bacteria and Archaea share a larger number of selenoprotein families if compared to Eukarya (Mariotti et al., 2016).

**FIGURE 2:**
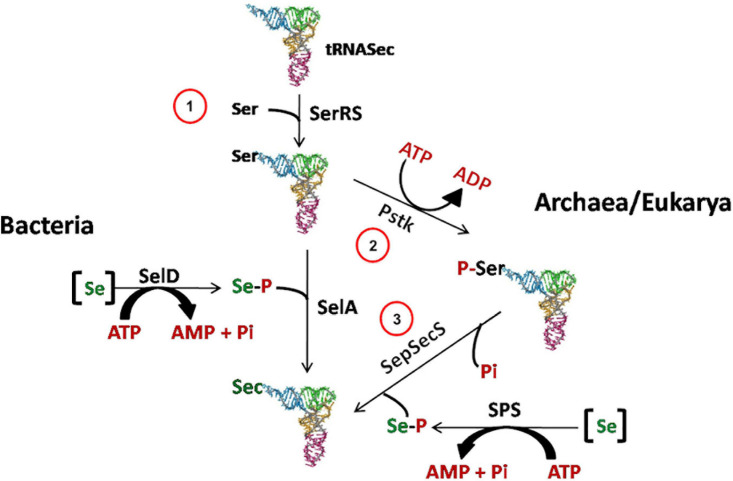
Sec biosynthesis in the three domains of life. In Archaea, as well as in Eukarya, Sec is synthesized in three steps. First (1), SerRS acylates tRNASec with serine to generate Ser-tRNASec. Then (2) PSTK forms Sep-tRNASec, which is converted to Sec-tRNASec by SepSecS in the presence of selenophosphate produced by selenophosphate synthetase (SPS) (3). -[Se]: reduced Se species; -SerRS: seryl-tRNA synthetase; -SelD/SPS: selenophosphate synthetase; -SelA: bacterial Sec synthase; -PstK: seryl-tRNASec kinase; -SepSecS: O-phosphoseryl-tRNA:selenocysteyl-tRNA synthase.

In Archaea, Bacteria and Eukarya, Sec is synthesized in a tRNA-bound fashion, although the mechanisms of Sec synthesis and insertion show differences in the three domains of life (Figure 2). While archaea and eukaryotes first catalyze the synthesis of phospho-Ser with the protein phosphoseryl-tRNASec kinase (PSTK), and then convert it to Sec, bacteria directly synthesize Sec from Ser (For a review see Rother and Quitzke, 2018).

**FIGURE 3:**
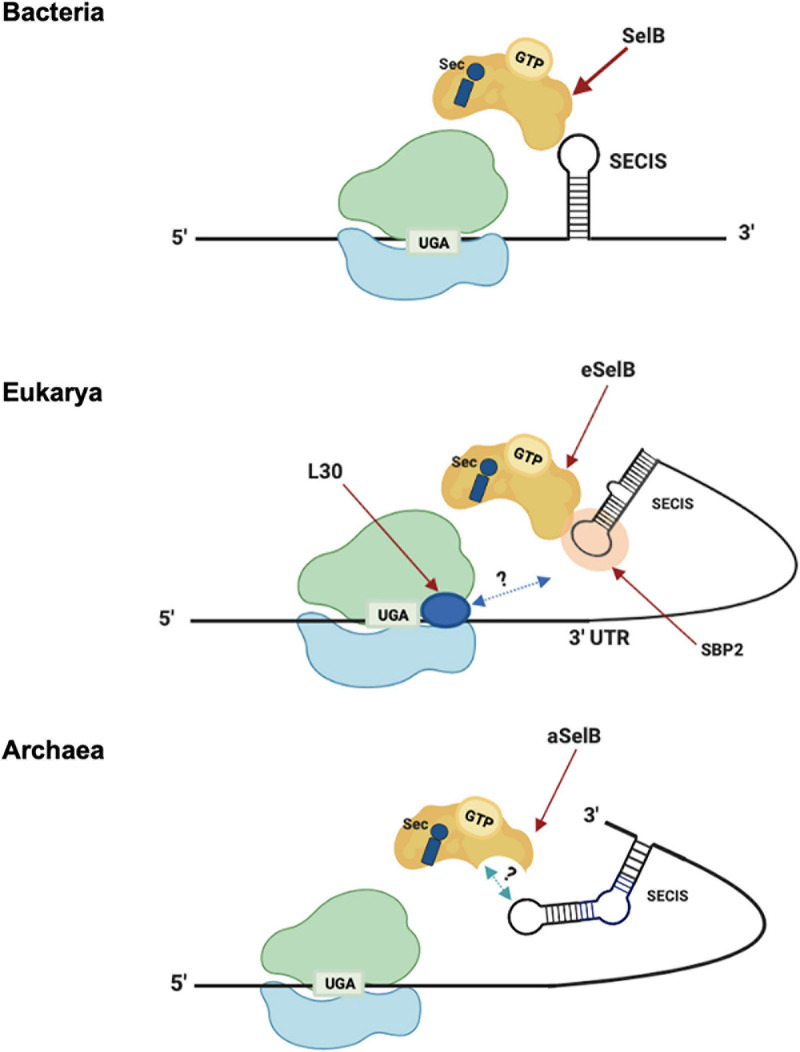
Sec translation in the three domains. Model of Sec incorporation in Bacteria (top), Eukarya (middle), and Archaea (bottom). -3′-UTR: 3′-untranslated region; -L30: ribosomal protein L30; -SBP2: SECIS-binding protein 2; -SECIS: Sec insertion sequence; -SelB/aSelB/eSelB: Sec-specific elongation factor.

The insertion of Sec is driven by specific signals found in the selenoprotein gene transcripts in *cis*. These signals are RNA structures, named SECIS (SElenoCysteine Insertion Sequence) elements (Berry et al., 1991; Figure 3). In response to those signals, the specific elongation factor SelB replaces the standard EF-Tu and recruits the Sec-tRNA, promoting the insertion of Sec residues in a specific UGA (Hatfield and Gladyshev, 2002; Mariotti et al., 2016). Interestingly, SECIS elements do not share similarity in sequence or structure between the three domains of life (Krol, 2002). In bacteria, the SECIS element (bSECIS) is a stem–loop structure located within the coding sequence, immediately downstream of the recoded UGA. The bSECIS is bound directly by the elongation factor bSelB through its C-terminal extension (see above) (Figure 3). The eukaryotic SECIS elements are, instead, located in the 3′ UTR of selenoprotein transcripts and they do not interact directly with eSelB, but though the SECIS Binding Protein 2 SBP2 (Tujebajeva et al., 2000; Fletcher et al., 2001). In addition, it has been found that other factors are involved in eukaryal Sec insertion, as the ribosomal protein L30 (Chavatte et al., 2005).

The archaeal versions of SECIS (aSECIS) are characterized by two stems separated by an invariant asymmetric bulge (Krol, 2002; Kryukov and Gladyshev, 2004; Stock and Rother, 2009) and are normally located in the 3′ UTR of selenoprotein coding mRNA, with a single documented exception (Wilting et al., 1997). To date, no aSECIS binding factors have been identified. The SBP2 homolog has never been observed in archaea, and it has been shown that the archaeal SelB does not bind aSECIS elements (Mariotti et al., 2016). Thus, it has been proposed that the eukaryal Sec decoding mechanism, in which SBP2 is a key factor, evolved after the transition from archaeal to eukaryotic-like SECIS elements (Stock and Rother, 2009). From an evolutionary point of view, the distribution of selenoproteins in living organisms is consistent with the phylogenetic relationship between the organisms in the three domains of life (Mariotti et al., 2015). In addition, considering the clear homology between the key factors involved in the Sec pathway (tRNAsec, SelB, and the selenophosphate synthetase SPS/SelD) (Santesmasses et al., 2017), it was highlighted that it is very likely that this pathway originated only once in the history of life and was already present in the Last Universal Common Ancestor (LUCA) (Mariotti et al., 2016). However, the presence of this pathway in different living organisms appears to be very dynamic, showing both clear events of horizontal gene transfer and independent loss in many lineages (Zhang et al., 2006; Lobanov et al., 2008; Mariotti et al., 2015).

Selenoproteins are a quite rare feature among the Archaea. Sec was found in formate dehydrogenase, formylmethanofuran dehydrogenase, F_420_ reducing and non-reducing hydrogenases, HesB-like protein and heterodisulfide reductases (Kryukov and Gladyshev, 2004; Stock et al., 2010). For a detailed list of putative and known archaeal selenoproteins and their properties see Rother and Quitzke (2018). Interestingly, genes encoding selenoproteins, belonging to different families, and the full set of genes encoding for the key factors involved in the Sec pathway, have been found in *Lokiarchaeota* (Spang et al., 2015), considered the closest cultured archaeal relative of eukaryotes (Mariotti et al., 2016). The selenoprotein families identified in *Lokiarchaeota* were previously reported in other archaeal lineages (Stock and Rother, 2009), with the exception of the thioredoxin-like superfamily, found by bioinformatic analysis, both in bacteria (Zhang and Gladyshev, 2008) and eukaryotes (Jiang et al., 2012; Mariotti et al., 2013). Moreover, although the selenoprotein genes in Lokiarchaeota are typical of archaea, they possess conserved RNA structures similar to eukaryotic SECIS elements. This finding is the basis of a new theory proposing that eukaryotes have not reinvented the mechanism of insertion of the Sec as previously proposed, but rather that the Sec pathway has passed vertically from Archaea to Eukarya (Rother and Quitzke, 2018).

### The 22nd Amino Acid: Pyrrolysine

Pyrrolysine (Pyl) was identified in 2002 as the 22nd proteinogenic amino acid (Hao et al., 2002; Srinivasan et al., 2002). From a biochemical perspective, Pyl is a typical L-lysine amino acid to which a pyrrole ring is branched on the lateral chain through an amide bond. This chemical modification is different from those present in other L-lysine derivatives found in some proteins from archaea like hypusine or methyllysine (Eichler and Adams, 2005). In fact, while in hypusine and methyllysine the modifications originate from post-translational events, Pyl is translationally incorporated (for a review see Brugère et al., 2018). This unusual and highly specialized amino acid is found in a small number of archaea able to metabolize methylamine as well as a few bacteria. The first hint of the presence of pyrrolysine (Pyl) has been reported in several Methanosarcina species with a total of 21 genes of mono, di-, and trimethylamine methyltransferases (MtmB, MtbB, and MttB, respectively) showing an in-frame amber UAG codon (James et al., 2001). Initially, the amino acid inserted into the UAG codon was identified as a lysine. Later, the three-dimensional structure resolution of the enzyme MtmB allowed to demonstrate that the amino acid was a Pyl. Furthermore, the identification of a specific tRNA for Pyl confirmed the hypothesis that Pyl is inserted into proteins during translation by a mechanism of recoding (Hao et al., 2002; Srinivasan et al., 2002). From these preliminary discoveries, several new pieces of information have been collected that have allowed to define the key factors involved in the biosynthesis and insertion of Pyl, the molecular mechanism underlying this recoding mechanism, its distribution and evolution, and the catalytic role of this amino acid.

**FIGURE 4:**
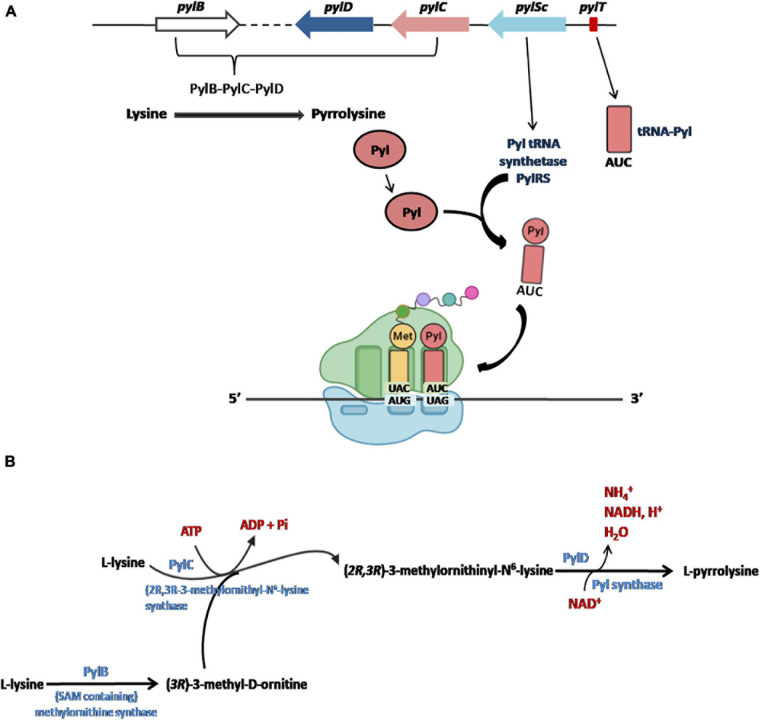
The Pyl insertion system **(A)**. Pyl, synthesized by pylB, pylD, pylC, is charged on a specific tRNA (encoded by pylT) whose anticodon AUC recognizes UAG codons in a specific reaction catalyzed by PylRS (encoded PylSc). See text for details. Figures arranged from Brugère et al. (2018). Biosynthesis of Pyl **(B)**. The complete biosynthesis pathway of L-pyrrolysine from two lysines catalyzed by PylB, PylC and PylD.

The five Pyl genes involved in the biosynthesis and genetic encoding of Pyl are pylTSBCD (Srinivasan et al., 2002; Krzycki, 2004; Zhang et al., 2005; Longstaff et al., 2007) and, in most cases, they are organized in an operon-like structure as shown in Figure 4A. The Pyl genes have been found in bacterial and archaeal genomes and are usually clustered near the genes encoding the methylamine methyltransferases and other genes involved in methylamine metabolism (for a detailed description of the genomic contexts of Pyl-related genes see Gaston et al., 2011).

Initially, it had been proposed that the synthesis of Pyl took place from the lysyl-tRNA-Pyl (Srinivasan et al., 2002; Polycarpo et al., 2003), similarly to how it occurs for the synthesis of the Sec starting from the seryl-tRNA (Figure 2; Yoshizawa and Bock, 2009; Rother and Krzycki, 2010). However, it is now well documented that Pyl is synthesized by the enzymes PylB, PylC, and PylD from two equivalents of lysine. The two other genes of the Pyl system, *pylT* and *pylS*, encode, respectively, for the tRNAPyl, whose anticodon is complementary to the UAG codon, and the subunit of the tRNAPyl synthetase which directly esterified Pyl to the 3′-hydroxyl of tRNAPyl, clearly demonstrating that Pyl is made independently of tRNAPyl (Figure 4A; Blight et al., 2004; Polycarpo et al., 2004; Nozawa et al., 2009; Gaston et al., 2011; Tharp et al., 2018). The complete pathway of biosynthesis of Pyl is reported in Figure 4B (for a review see Brugère et al., 2018).

Although possible sequences that regulate Pyl (named PYLIS by analogy to the SECIS sequences, see above) were initially postulated (Namy et al., 2007), bioinformatic (Zhang et al., 2005) and biochemical studies have shown that no *cis* element is found or required in *E. coli* for the recoding of the UAG stop codon into Pyl (Longstaff et al., 2007; Namy et al., 2007). It follows that there is no specific context in the mRNA driving the recoding event, therefore it was proposed that Pyl insertion relies only on the competition between release factors and the tRNA-Pyl during translation. However, how the cell prevents all stop codons from being recoded is still to be elucidated, especially considering that *cis* signals have not been found. Interestingly, it has been reported that in the clostridial *Acetohalobium arabaticum*, UAG specifies Pyl only when the cells are grown in trimethylamine, while, when the cells are grown on pyruvate as a carbon source, UAG only specifies termination (Prat et al., 2012). Thus, this result indicates that Pyl insertion is regulated in specific physiological conditions and could suggest the presence of a *trans*-acting regulation factor expressed only in particular conditions which must still be identified.

Pyl is found in all methanogen methylamine methyltransferase genes and in some cases the readthrough efficiency of the UAG codon is as high as 97%. In these enzymes Pyl is always present in the active site, capturing methylamines before transferring one methyl group to a Co(I)-corrinoid cofactor of an associated protein (MtmC/MtbC/MttC) (Hao et al., 2002), suggesting that its role is fundamental for methylamine metabolism. More recently, it has been reported that natural MttB analogs without Pyl found in *Desulfitobacterium hafniense* has a glycine betaine methyltransferase activity (Ticak et al., 2014), confirming that methyltransferases containing Pyl are related to methylamines metabolism. The only other known Pyl-containing proteins are some transposases (Zhang et al., 2005), and a tRNAHis-guanylyltransferase Thg1 (Heinemann et al., 2009) both present in a subset of Methanosarcinales.

In archaea, pyl genes were initially identified in anaerobic methanogens living in environments where methylamines are available, namely, several Methanosarcinales (Deppenmeier et al., 2002; Galagan et al., 2002; Maeder et al., 2006), in *Methanococcus burtonii* (psychrophile) (Goodchild et al., 2004), and in *Methanoalophilus mahii and Methanohalobium evestigatum* (halophiles) (Rother and Krzycki, 2010; Gaston et al., 2011). More recently, the genes for the synthesis and encoding of Pyl were identified in several new lineages of methanogens, discovered by metagenomic approaches and distantly related to those mentioned above, in which the methanogenesis is dependent on methyl-compounds (Borrel et al., 2013; Evans et al., 2015; Petitjean et al., 2015; Nobu et al., 2016; Vanwonterghem et al., 2016; Sorokin et al., 2017). Pyl-containing methyltransferases needed for methylamine utilization are always present in these new lineages of methanogens that contain the Pyl system, strengthening the hypothesis that the Pyl system is dedicated to the incorporation of Pyl in these methyltransferases, and thus associated to methylamine utilization. Methanohalophilus, in which the Pyl-containing methyltransferases are absent (Fricke et al., 2006) are also lacking the Pyl system, suggesting that this recoding mechanism is linked to methylamine methyltransferases rather than to archaea performing methanogenesis based on methyl-compounds. In addition, it has been found that uncultured sugar-fermenters of the candidate division of Persephonarchaea, thriving in a hypersaline environment, harbor a complete set of genes for Pyl synthesis and *mtmB*, *mtbB*, and *mttB* genes (Guan et al., 2017). The components of the Pyl system in these archaea are phylogenetically related to those found in the bacteria *Acetohalobium arabaticum* who lives in the same environment, suggesting an event of horizontal gene transfer between these organisms (Guan et al., 2017). From its first discovery great advances have been made in understanding the role of this recoding event in archaea and allowing us to reveal that Pyl-system has a wide distribution and is not necessarily associated with methanogenesis in this domain of life (Brugère et al., 2018).

There are several hypotheses for the emergence of the Pyl system in living organisms. Among the others, one of the most recent, and strongly supported by current data, postulated that the Pyl trait is very ancient and probably only emerged once after LUCA and was linked to methanogenesis. The trait could have then evolved and preserved in organisms for which methylamine metabolism was fundamental to survive and could have been further spread across the bacterial and archaeal domains by horizontal gene transfer (Brugère et al., 2018).

## Programmed Ribosomal Frameshifting

During standard mRNA translation the ribosome initiates protein synthesis at a start codon and moves by decoding three nucleotides at a time until it reaches a stop codon where translation is terminated. However, in some cases the ribosomes switch to an alternative reading frame on the mRNA by determining a translational slippage in the +1 or –1 direction (Farabaugh, 1996; Gesteland and Atkins, 1996; Figure 1). In contrast to spontaneous frameshifting, which produces non-functional polypeptides, PRF is generally in competition with standard decoding and typically leads to the synthesis of a functional polypeptide from an alternative frame with efficiencies varying from very low to as high as 80% (Tsuchihashi and Brown, 1992; Atkins et al., 2009). At the functional level there are two more common classes of regulation of PRF. In a first class, often termed ‘set ratio’ frameshifting, the proportion of ribosomes that shift frame is constant, thereby generating an extra N-terminally coincident product. In a second class, frameshift efficiency is dependent by the level of translation initiation or responsive to a trans-acting factor. Here, frameshifting, acting as a sensor and/or effector has a regulatory function, allows the synthesis of a functional trans-frame encoded product or alters mRNA half-life (Atkins et al., 2016). It has been well demonstrated that in eukaryotes, PRF can regulate the stability of an mRNA. In fact, it has been seen that following a PRF event, the ribosomes encounter a stop codon in the new reading frame that activates the nonsense-mediated decay pathway (Belew et al., 2014).

The PRF has been studied extensively in viruses, where –1 PRF plays an important role in viral propagation by modulating synthesis of viral proteins in specific stoichiometric ratios (Jacks and Varmus. 1985; Plant et al., 2010). The use of a –1 PRF mechanism for the expression of a viral gene was first identified in the Rous sarcoma virus (Jacks and Varmus, 1985). To date, it is well known that, for example, all coronaviruses utilize –1 PRF to control the relative expression of their proteins. In general, the early translated viral proteins are involved in neutralizing the host cellular immune response (ORF1a) and in genome replication and RNA synthesis (ORF1b). ORF1b is in the –1 reading frame with respect to ORF1a, and all coronaviruses, as well as SARS-CoV-2, utilize –1 PRF as a mean to synthesize the ORF2 encoded proteins (Kelly et al., 2020). PRF is well documented in retrotransposons and insertion elements too, while it is less common in cellular genes. Among the chromosomal genes, the best studied examples are the Antizyme (Matsufuji et al., 1995) in which + 1 PRF frameshifting functions both as a sensor of the polyamine levels and as an effector of a self-regulating circuit from yeasts to mammals. In the bacterial DNA polymerase, γ and τ subunits are produced in 1:1 molar ratio by –1 PRF from *dnaX* gene (Tsuchihashi and Kornberg, 1990; Mangold, 2005; Chen et al., 2014). For a comprehensive review on the genes expressed by PRF in Bacteria, Eukarya and viruses see Atkins et al. (2016); Rodnina et al. (2020). Among PRF, –1 frameshifting is more widespread with examples in all three domains of life (Luthi et al., 1990; Tsuchihashi and Kornberg, 1990; Cobucci-Ponzano et al., 2006; Wills et al., 2006; Belew et al., 2014), many of which are phylogenetically conserved.

As stated above, –1 PRF is generally in competition with standard decoding but it is facilitated by two regulatory elements in the mRNA sequence, a slippery site, where the transition to the –1 frame takes place, and a secondary structure element (a pseudoknot, a steam and loop or a kissing loop) at a defined distance of 5 to 9 nucleotides from the slippery site (Brierley et al., 1992, 2010; Atkinson et al., 1997; Lin et al., 2012; Choi et al., 2020). The slippery site, usually in the form of a heptanucleotide sequence X-XXY-YYZ, in which X can be any base, Y is usually A or U, and Z is any base but G (codons are shown in the 0 reading frame), allows for base pairing between the tRNA anticodon and the mRNA codon after shifting into the –1 reading frame. Prokaryotic frameshifting sites may contain additional stimulatory elements, such as an internal Shine-Dalgarno (SD)-like sequence upstream of the slippery site (Larsen et al., 1997; Choi et al., 2020) or tandem rare codons (Caliskan et al., 2017) both with the function of slowing down the translating ribosome and increasing the frameshifting efficiency. –1 PRF can be also facilitated by miRNAs binding as reported in the human mRNA encoding the HIV-1 co-receptor CCR5 (Belew et al., 2014), or proteins, as reported in some viruses (Kobayashi et al., 2010; Napthine et al., 2017; Wang et al., 2019) to the sequence following the slippery site.

Detailed studies on the molecular mechanism by which –1 PRF occurs have only recently been reported. These studies suggest that the molecular mechanisms are mainly two and depend on the availability of the aa-tRNAs of the codons in the slippery sequence (Namy et al., 2006; Chen et al., 2013, 2014; Caliskan et al., 2014, 2017; Kim et al., 2014; Yan et al., 2015; Korniy et al., 2019a, b). When the tRNAs reading the slippery sequence codons are abundant, –1 PRF occurs at the late stage of translocation, with two tRNAs moving through the ribosome, and requires the presence of the stimulatory element within the mRNA sequence. By contrast, in conditions in which aa-tRNAs are limited, the –1 PRF occurs via one-tRNA slippage of the P-site tRNA, when the A site is vacant, and its efficiency is independent of the stimulatory element within the mRNA sequence. This latter mechanism is often called “hungry” frameshifting, because it can be triggered by aa-tRNA limitation due to starvation (Gallant and Lindsley, 1992; Olubajo and Taylor, 2005; Temperley et al., 2010) (see below).

**FIGURE 5:**
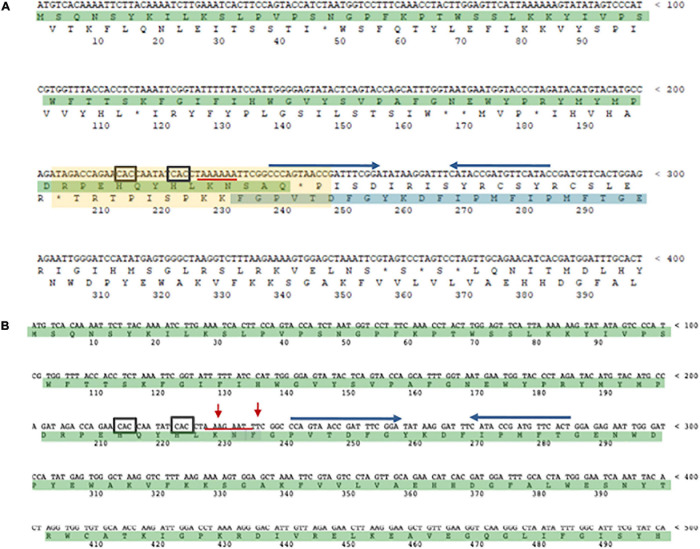
The α-L-fucosidase gene. **(A)** The N-terminal SSO11867 ORF (highlighted in green) is in the zero frame, the C-terminal SSO3060 ORF (highlighted in blue), for which only a fragment is shown, is in the –1 frame. The 40 bp region of overlap bertween the two ORFs is indicated with a light yellow rectangle. The slippery heptameric sequence is underlined with a red line. The rare codons CAC are indicated with a black square. The putative stem and loop region is indicated with blu arrows. **(B)** *framefucA* mutant gene (only a fragment is shown). The red arrows indicate the mutated nucleotides in the slippery sequence.

**FIGURE 6:**
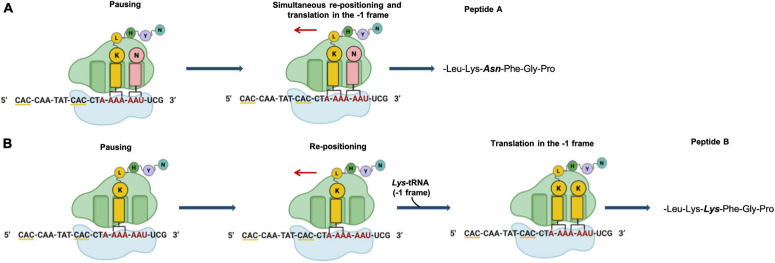
Putative mechanism of programmed –1 frameshifting. **(A)** Simultaneous P- and A-site slippage; **(B)** P-site slippage. The slippery heptameric sequence is indicated in red. Rare codons are underlined with yellow line.

In Archaea only one case of –1 PRF has been reported (Cobucci-Ponzano et al., 2006). In the thermoacidophilic archaeon *Saccharolobus solfataricus* (formerly *Sulfolobus solfataricus*) (Sakai and Kurosawa, 2018) strain P2 the *fucA1* gene was found to be organized in two open reading frames (ORFs) SSO11867 and SSO3060 of 81 and 426 amino acids, respectively, which are separated by a –1 frameshifting in a 40 bases overlap. These ORFs encode, respectively, for the N- and C-terminal part of a α-*L*-fucosidase. The overlap region between the two ORFs had the characteristic features of the genes expressed by –1 PRF, including a heptanucleotide A-AAA-AAT (codons are shown in the zero frame), flanked by a putative stem and loop and the tandem rare codons CAC (Figure 5). To test if these gene fragments could lead to a functional enzyme, a full-length gene, named *framefucA*, was produced by inserting specific site-directed mutations in the *fucA1* gene, exactly in the position predicted by –1 PRF. In this way, the poly-A sequence of the *slippery* site was disrupted and a T nucleotide was inserted to restore a single reading frame between the two ORFs (Cobucci-Ponzano et al., 2003a). The *framefucA* mutant encoded for a polypeptide of 495 amino acids, that, remarkably, in recombinant form produced a fully functional α-*L*-fucosidase, named Ssα-fuc, which was thermophilic, thermostable and had an unusual non-americ structure (Cobucci-Ponzano et al., 2003b, 2005a; Rosano et al., 2004). The full-length protein FucA was expressed by –1 PRF in both *E. coli* and *S. solfataricus* showing for the first time that this kind of recoding is present in Archaea (Cobucci-Ponzano et al., 2006). The observation that the *fucA1* interrupted gene directed the expression of low α-*L*-fucosidase activity in *E. coli* led to the isolation and characterization of the polypeptides expressed in the recombinant form demonstrating that the *fucA1* gene produced in *E. coli* a mixture of two full-length polypeptides, both functional, with a total efficiency of about 5% (Xu et al., 2004; Cobucci-Ponzano et al., 2006). The identification of these polypeptides indicated that the translational recoding of *fucA1* might occur in two ways, at least in *E. coli* (Figure 6): a simultaneous backward slippage of the ribosome when both the P- and the A-site tRNAs are occupied (Figure 6A) and/or the repositioning of the ribosome in the –1 frame when only the P-site tRNA is bound (Figure 6B) (Cobucci-Ponzano et al., 2006). The analysis of *fucA1* –1 PRF in *S. solfataricus* by *in vitro* translation revealed that only the *wild type* slippery sequence led to the translation of a full-length product with good efficiency (about 10%), demonstrating that this process occurred in archaea (Cobucci-Ponzano et al., 2006). *In vivo*, full-length polypeptides from *fucA1* were identified in *S. solfataricus* extracts, and reverse real-time PCR experiments and specific enzymatic assays confirmed that this enzyme was functionally expressed though at very low levels.

Although these studies produced evidence that –1 PRF is present in archaea, several questions remain unanswered: to date, it is still not known why the translational of *fucA1* in *S. solfataricus* is regulated by recoding, and if other genes are expressed by this mechanism in this or other archaea. However, since there are no α-*L*-fucosidase genes regulated by PRF in Bacteria and Eukarya, it has been suggested that this sophisticated mechanism of translational regulation preexisted in *S. solfataricus* and it was applied to the α-*L*-fucosidase gene for physiological reasons. Very recently, it has been found that *fucA1* mRNA increases by 10 *fold* after *S. solfataricus* undergoes cold shock and in *S. solfataricus* cells grown in minimal medium containing the oligosaccharides of the hemicellulose xyloglucan (De Lise et al., 2021). Furthermore, this α-*L*-fucosidase has been shown to cooperate with other glycoside hydrolases from *S. solfataricus* for the hydrolysis of fucosylated xyloglucan oligosaccharides by removing the fucose moieties from this substrate with high efficiency *in vitro* (Curci et al., 2021). These new results will certainly need to be explored and could be of great help in understanding what the function of this enzyme is *in vivo*, and why its expression is regulated by 1- PRF.

Genomic sequencing showed that the *fucA1* gene was also present in other archaea, all belonging to Crenarchaeota (for the compilation of these genes, see the Carbohydrate Active enZyme database^2^). The α-L-fucosidases from Sulfolobales showed 96% amino acid sequence identity and are all full length with the exception of the *S. solfataricus*, strain 98/2 which presented the frameshifting in the same position as the gene from strain P2. However, all Sulfolobales genes showed 100% DNA sequence identity in the region of the frameshifting, maintaining the rare codon, the slippery sequence, in which the stretch of A is shortened by one nucleotide in full-length genes, and the putative stem loop. On the contrary, the slippery sequence is not conserved in full-length α-*L*-fucosidase homologs from *I. aggregans* and *C. maquilingensis*. Remarkably, full-length α-*L*-fucosidases, in the region of the slippery sequence, have the same Lys or Asn amino acids observed in the full- length product of the wild-type interrupted *fucA* (Cobucci-Ponzano et al., 2006).

More recently, PRF events have been reported in some archaeal viruses. In particular, –1 PRF seems to be used by the siphoviruses tailed virus 1 (HVTV-1) and three viruses (HCTV-1,2 and 5) that infect halophilic archaea, while an event of +1 PRF appears to be present in the haloarchaeal myovirus tailed virus 2 (HSTV-2) (Pietila et al., 2013; Sencilo et al., 2013). In addition, it has been suggested that a frameshifting is presumably involved in the synthesis of magnesium chelatase from the archaea *Methanocaldococcus* and *Methanococcus* (Antonov et al., 2013b). Unfortunately, genes with frameshifts could be difficult to annotate by standard procedures and often might be annotated as two separate adjacent hypothetical genes (Antonov et al., 2013b). In recent years some bioinformatic tools have been developed with the aim of identifying possible genes regulated by frameshifting (Antonov and Borodovsky, 2010; Antonov et al., 2013a). However, none of these have been systematically tested on Archaea and it would be very useful to know whether the parameters used allow to identify possible genes regulated by frameshifting in this domain of life.

## Conclusion

The identification of novel genes whose expression could be regulated translational recoding is not easy, either because disrupted genes are commonly considered non-functional pseudogenes or because technical limitations, and this is particularly true for Archaea for which molecular biology tools are still to be completely developed. Non-functional pseudogenes are present in organisms from all the living domains, though in some cases they have been demonstrated to be useful for an organism’s survival and adaptation to particular environmental changes (Harrison and Gerstein, 2002; Balakirev and Ayala, 2003; Hirotsune et al., 2003). In Archaea, 15 different species have been bioinformatically analyzed revealing a high number of predicted pseudogenes, the highest of which (8.6% of the annotated protein coding sequences) being in *S. solfataricus*. The expression of these genes has not been tested but, remarkably, all the frameshifts occurred in A/T rich DNA tracts resembling the slippery sequences regulating –1 PRF in *cis* (van Passel et al., 2007). In addition, a different bioinformatic analysis of other 16 Archaea genomes, allowed to identify a large number of disrupted genes, some of which resulted to be functional, as demonstrated by a high throughput proteomic analysis and functional characterization of some of them from *S. solfataricus* strain P2 (Cobucci-Ponzano et al., 2010). Interestingly one of the interrupted gene whose expression could be regulated by –1 PRF is the putative universal translation initiation factor SUI-1/aIF1 (Cobucci-Ponzano et al., 2010). This protein is essential in yeast forming the translation initiation complex and monitoring the maintenance of the correct translational reading frame in eukaryotes, such as it was suggested that it might govern programmed –1 frameshifting as a trans-acting factor (Cui et al., 1998; Kyrpides and Woese, 1998). Similarly, *in vitro* experiments performed with *S. solfataricus* cell fractions showed that aIF1 promotes translation complex binding to the ribosome, promoting discrimination against non-canonical start codons and enhancing translation efficiency (Hasenöhrl et al., 2006 RNA.; 12: 674–682.; Hasenöhrl et al., 2009 RNA.; 15: 2288–2298.) In the genome annotation of *S. solfataricus*, P2 strain, this gene is reported as interrupted by –1 frameshfting, but, once re-sequenced, it was found to be full-length, suggesting a possible sequencing error (Hasenöhrl et al., 2006; Cobucci-Ponzano et al., 2010). However, a high-throughput proteomic analysis revealed the presence of two peptides, one deriving from the full-length gene and the other one deriving from the translation of the annotated interrupted gene by –1 PRF (Cobucci-Ponzano et al., 2010). These data merit further investigation and could be of some help to elucidate the possible mechanism of expression of this gene in *S. solfataricus* and to shed some light of its role *in vivo*.

It has been suggested that the flexibility of the genetic code decoding, typical of recoding mechanisms, is a trait selected during evolution that may increase microbial fitness under certain conditions (Ling et al., 2015). The majority of Archaea populate extreme environments, which are often spots (e.g., hydrothermal vents, solfataras, etc.) surrounded by environments with milder conditions and frequently subjected to sudden changes that greatly, and temporarily, modify the chemical-physical parameters to which microorganisms must adapt. It is tempting to speculate that in these extreme environments translational recoding could be a way to maintain in a latent state the expression of certain genes, and up- or down-regulate them under specific conditions. Another important aspect to be considered is related to the understanding of the molecular mechanisms that lead to the improved fitness as a result of genetic code variation (Ling et al., 2015). This fostered a new research area in engineering synthetic organisms with new genetic codes and non-canonical amino acids (for a review see Hoffman et al., 2018). These engineered synthetic organisms will be very important to study the physiological effect of genetic code evolution (Ling et al., 2015). Thus, the study of translational recoding in Archaea is particularly important for its possible implications in the evolution of the genetic code and the correlation between the flexibility of the genetic code decoding and improved fitness in extreme environments.

## Author Contributions

FDL and BC-P wrote the manuscript. AS, RI, NC, LM, MDF, and MM contributed to the article, edited English style, and approved the submitted version. MDF, MM, FDL, and BC-P edited the manuscript into its final format. All authors contributed to the article and approved the submitted version.

## Conflict of Interest

The authors declare that the research was conducted in the absence of any commercial or financial relationships that could be construed as a potential conflict of interest.

## Footnotes

1 http://recode.ucc.ie

2 http://www.cazy.org/
